# Linearity of Age at Cancer Onset Worldwide: 25-Year Population-Based Cancer Registry Study

**DOI:** 10.3390/cancers13215589

**Published:** 2021-11-08

**Authors:** Ettore Bidoli, Elda Lamaj, Tiziana Angelin, Ornella Forgiarini, Emilia De Santis, Diego Serraino

**Affiliations:** 1 Unit of Cancer Epidemiology, Centro di Riferimento Oncologico di Aviano (CRO) IRCCS, via Gallini 2, 33081 Aviano, Italy; tangelin@cro.it (T.A.); serrainod@cro.it (D.S.); 2 Friuli Venezia Giulia Cancer Registry, Centro di Riferimento Oncologico di Aviano (CRO) IRCCS, via Gallini 2, 33081 Aviano, Italy; lamajelda@yahoo.it (E.L.); ornellaforgiarini@tiscali.it (O.F.); emiliadesantis1@gmail.com (E.D.S.)

**Keywords:** cancer, incidence, age at onset, population pyramid, worldwide, 25-year

## Abstract

**Simple Summary:**

The age at cancer onset varies worldwide and is intuitively linked to the age structure of the population originating the cases. The exact amount by which age explains this pattern has been estimated for breast cancer to give clues about the 10-year difference in age at cancer onset between low/middle and high income countries. The age contribution for 20 selected cancer types was calculated, using cancer incidence data from all worldwide cancer registries, through linear regression models. For all cancers but skin, age explained 56% of the pattern in men and 65% in women. The percentages varied by cancer types. Since age at cancer onset is embedded with the age structure of the underlying population, it is possible to avoid misinterpretations of a high frequency of cancer onset in specific age groups by inspecting the age pyramid of the population to check for the plausibility of the excess.

**Abstract:**

Background The linear association between median age at cancer onset and median age of the underlying population has been described only for breast cancer. We quantified the shape and strength of such association for 20 cancer types using data from all population-based cancer registries (CRs) worldwide. Methods The patients’ median age at cancer onset and of the underlying population were extracted from all CRs listed in volumes VI (1983–1987 years) and XI (2008–2012 years) of Cancer Incidence in Five Continents. The association was assessed at cross-sectional level by linear regression models and longitudinally considering only the long-standing CRs active throughout the study period (i.e., 25-year span). Results During 2008–2012, each one-year increase in median population ageing was associated in men with a nearly half year increase of median age at onset of all cancers, but skin; and a 2/3 year increase in women. Variance explained by linear model was around 60%. In long-standing CRs a decrease in median age at cancer onset was observed for prostate and cervical cancers throughout the 25-year span. Conclusions Population ageing reflected 60% of the median age at cancer onset. Misinterpretation of peaks of cancer incidence in specific age groups may be avoided by examining population pyramids.

## 1. Introduction

The median age at cancer onset varies worldwide across populations [1,2]. For instance, it has been observed that the median age at onset of female breast cancer (BC) was a decade higher in high income countries than in low/middle income countries (LMIC). This observation suggested lowering the age of entry into mass BC screening programs in LMIC [3]. However, the variation in median age at cancer onset is intuitively linked to the age composition of the underlying populations, like for many forms of cancer, age-specific incidence rates increase exponentially with ageing [4,5]. The strength and the shape of the association of the age at cancer onset with the age structure of the corresponding population has been described thoroughly only for BC [6,7]. Specifically, each one-year increase in median population ageing is linearly associated with a nearly ½ year increase of median age at BC onset with an explained 42% variance [7]. Thus, once the median age of a population is known, it is possible to estimate the median population age at BC onset. However, there is not much evidence to what amount age explains this association in other cancer sites or the shape of this association. The knowledge of this quantity may help interpreting high frequencies of cancer onset in specific age groups worldwide.

The age structure of incident cancer cases and of the population can be summarized by calculating a measure of central tendency of age, like the median age [8,9]. Thus, the population pyramids of younger populations characterized by a cone-shaped pattern will present a lower median age than populations with an urn shaped pattern (older ones) that will present a higher median age. Consequently, the worldwide impact of population ageing on age at cancer onset can be visualized and quantified by means of two approaches, as previously done for BC [7].

To address this issue systematically, we extracted all available worldwide cancer incidence data among the cancer registries (CRs) listed by International Agency for Research on Cancer (IARC). For 20 incident cancer sites or groups we described and quantified the association between the median age at onset and the median age of the underlying population at risk. The populations were disentangled as in our previous paper in three main geographical areas [7]. Two periods of registration (1983–1987, and 2008–2012) were considered. As regards the cross-sectional approach, incidence data from all available CRs were analyzed. Conversely, for the longitudinal approach incidence data were restricted to CRs that collected data throughout the study period (i.e., over a 25-year span).

## 2. Materials and Methods

### 2.1. Data Sources

An observational study was conducted using all reported incident cancer cases worldwide, occurred during two separate periods, i.e., 1983–1987 and 2008–2012. Study design and inclusion criteria have been described previously [7]. Briefly, we extracted publicly available data from two volumes (i.e., VI and XI) of Cancer Incidence in Five Continents [10,11]. Each volume collected cancer incidence data from all population-based, country-specific, or region-specific high-quality CRs located across the world. Data were disentangled by age (quinquennia), sex, and population (or ethnicities). Generally, the volumes provided data for a quinquenium from each CR. Since not all CRs were able to provide data for a complete quinquennium, a three-year consecutive series was the minimum inclusion requirement [11]. To avoid data duplicates, or the use of estimates of cancer incidence, we excluded the CRs derived by summing data from multiple populations, multiple CRs, or obtained from regional cancer estimations. The number of these unique populations increased from 160 in 1983–1987 to 393 in 2008–2012. Moreover, 84 CRs were listed in both VI and XI volumes. These CRs that gathered data in an approximately 25-year span were herein defined long-standing CRs.

The datasets included in the volumes VI and XI followed the evolution of the International Classification of Diseases (ICD), i.e., 9th and 10th revision, respectively [https://ci5.iarc, accessed on 28 October 2021]. This collection of datasets fully complied with international standards of cancer coding, allowing comparisons across calendar years. According to the ICD 10th revision, the 20 studied cancer sites or groups were: pharynx (C09–14), esophagus (C15), stomach (C16), colon, rectum and anus (C18–21), liver and intrahepatic bile ducts(C22), pancreas (C25), larynx (C32), lung (C33–34), breast for women (C50), cervix uteri (C53), prostate (C61), testis (C62), kidney and urinary tract (C64–66, C68), bladder (C67), Hodgkin disease (C81), non-Hodgkin lymphoma (C82–85, C96), multiple myeloma (C88–90), leukemia (C91–95), ill-defined or undefined sites, and all sites but non-melanoma skin cancer (C00–96, but C44).

The corresponding resident population disentangled by age (quinquennia), sex, calendar period, and CR was extracted from the same IARC database of the cases.

### 2.2. Statistical Analyses

For each CR, the age composition of incident cancer cases and the corresponding population structure was summarized by a measure of central tendency, the median age. The median age is the middle value dividing the population in two halves of equal size, and the higher the median age, the older the population [8,9,12]. In all IARC volumes, age is given in quinquennia (0–4, 5–9, …, 85+ years); thus, the median age was estimated [13]. Median age can be calculated from grouped data by means of a linear interpolation after taking into account the observations, the class size, the frequency, and the cumulative frequency [14]. We conducted a sensitivity analysis to test for potential differences between the estimated and the observed median age at cancer onset. To this end, we extracted cancer incidence for the period 2008–2012 in the Friuli Venezia Giulia CR, Italy (authorized by the CR Director: DS) [15]. The estimated median age of female BC was 65.5 years and the observed one was 65.6 years, while the estimated median age of male lung cancer was 72.8 years and the observed one was 72.8 years.

The first approach used in this study was a cross-sectional analysis. We visualized and quantified the association between the median age at cancer diagnosis and the median age of the corresponding population by including the 393 unique populations recorded in the XI volume (representing nearly one sixth of the world population located in 65 countries). CRs operating in low- and middle-income settings may face particular challenges in following international registration standards such as a lack of coverage by pathology laboratories or difficulty in accessing diagnosis records, which reduce the percentage of microscopically verified cases [11]. Similarly to our previous paper on BC [7], CRs were further analyzed by a priori selected geographical areas (Americas, Europe and Oceania, 285 CRs; Asia, 84 CRs; and Eastern Mediterranean and Africa, 24 CRs) as a geographic sensitivity analysis. In particular, for each cancer or group of cancers, the shape of the association between median age at cancer onset and age of the population was investigated by means of scatterplots. After visualizing the scatterplots, we assumed that the shape between the two groups of medians was linear. All statistical analyses were carried out by means of SAS (version 9.4 SAS Institute, Cary, NC, USA) using proc reg that produces by default a variety of diagnostic plots of the residuals versus the regressor. For instance, when considering all 393 CRs, in any cancer or group of cancers studied, the plots of residuals and studentized residuals versus the predicted values exhibited no specific pattern that contrasted the linear relationship hypothesized visually by examining the scatterplots. Moreover, the points of the plot of the dependent variable versus the predicted values lay along a 45-degree line, indicating that the model predicted the behavior of the median age at cancer onset variables. Thus, after examining the diagnostic plots, the analyses were considered acceptable to conclude in the linearity of the relationship studied. The strength of the association was measured by means of a simple linear regression model of the least square method. In the model, the median age of the population was the predictor variable while the median age at cancer onset was the dependent variable. The slope of the regression line measured the variation of the age at cancer onset corresponding to a one-year increase of the median age of the population. The percent of the variation of the median age at cancer onset that is explained through the linear relationship by the population ageing was quantified by means of a r^2^. Finally, an overall *F*-test verified if the r^2^ value was significantly different from zero. Linear regression models including all available CRs were calculated separately for the two IARC volumes. The linear slope and r^2^ was considered as statistically significant with a *p*-value < 0.05. We added the 95% predictive intervals to the scatterplots to display the range of values within which the median age at cancer onset was likely to fall given the median age of the underlying population in order to further check their linear relationship.

To support the strength of our study, we carried out two sensitivity analyses. Firstly, some datasets did not fully meet criteria [11] concerning quality and completeness of partly or all cancer sites, populations, or death certificates. Therefore, we recalculated the models for the 2008–2012 period excluding 109 CRs that did not fully meet these quality requirements (i.e., we analyzed 284 CRs instead of 393). No major differences were observed between the two types of models. Secondly, as it is known that age-specific rates of cancer increase exponentially with age [5], we verified whether an exponential relationship between age at BC onset and age of the population explained the variance better than a linear model. For instance, for all cancers but skin, the explained variance in men was 0.52 for the exponential model and 0.56 for the linear model. Therefore, the linear model was chosen for simplicity of graphical interpretation.

The second approach used in this study was a longitudinal analysis over a 25-year span. We considered exclusively the 84 long-standing CRs to evaluate the relationship between the 25-year population ageing and the age at onset of the studied cancers. A 2-point line graph displayed the median age at cancer onset according to the median age of the underlying population during the first (1983–1987) and the last periods (2008–2012) of observation. Due to the peculiar pattern of the graph in all cancers, but skin, a receiver operating characteristic (ROC) curve was calculated to identify the optimal cut-off of the median age in 1983–1987 that best predicted its decreasing after 25 years [16].

## 3. Results

**Figure 1:**
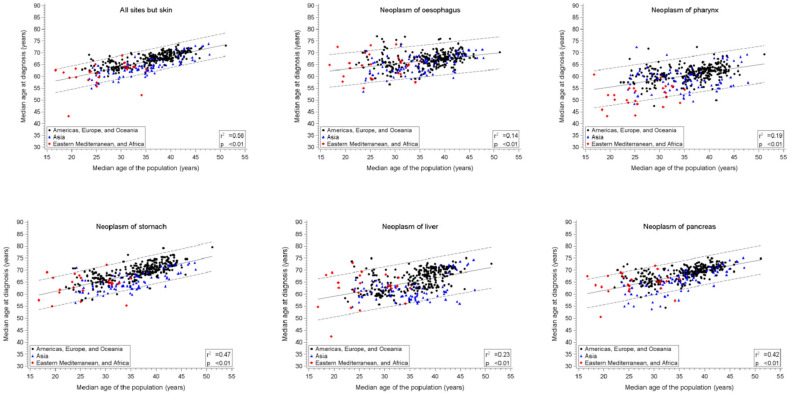

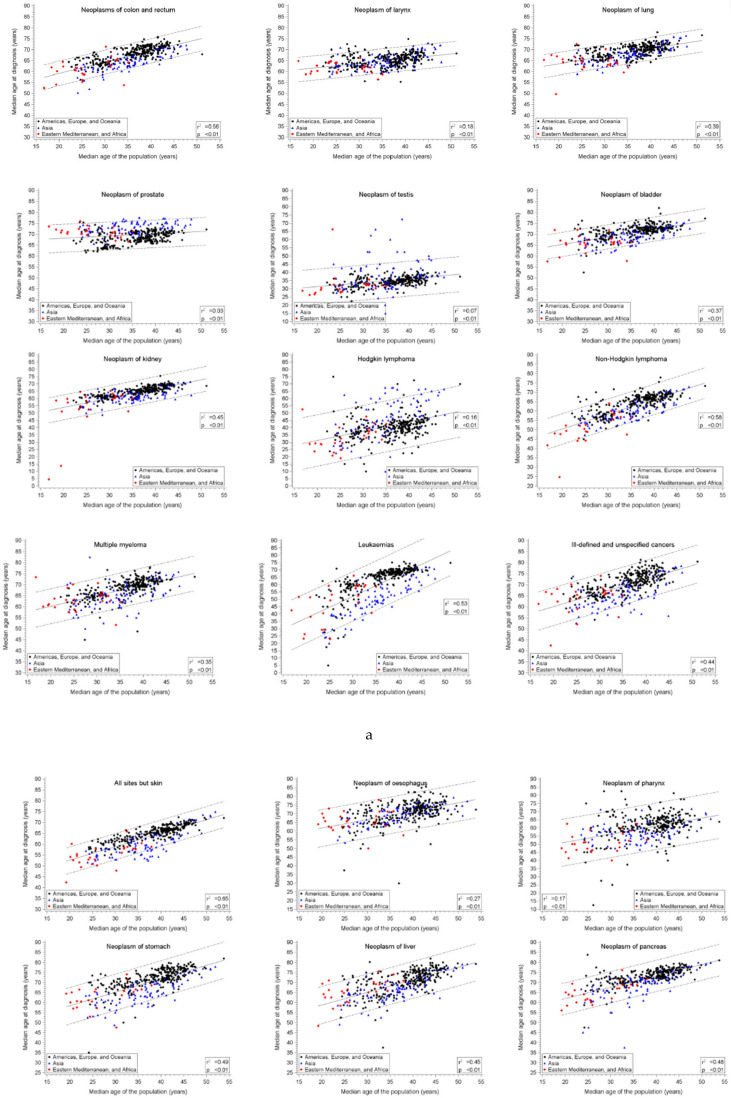

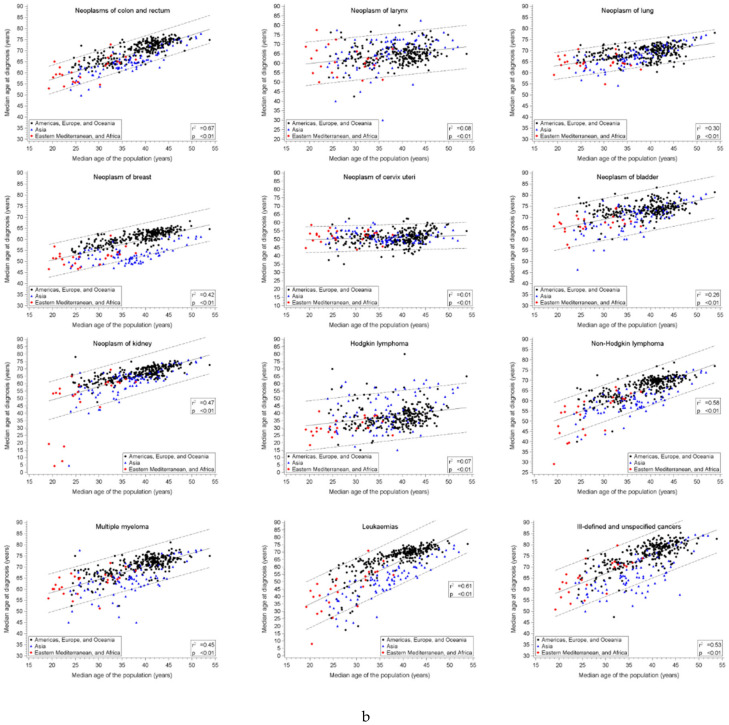
Scatter plots and linear regression with corresponding 95% predictive intervals, of the median age of the population (independent variable) vs. the median age of studied cancers cases in the populations recorded in all cancer registries listed in the XI volume of Cancer in Five Continents (period 2008–2012).(**a**) Males; (**b**) Females.

During the 2008–2012 period, about 69 million incident cancer cases were examined. When considering the long-standing CRs 4.4 million cases were studied. The association between the median age at onset of 20 cancer sites or groups and the median age of the corresponding population are represented by means of scatter plots and linear regressions for males (Figure 1a), and females (Figure 1b) for the period 2008–2012.

The intercept and the slope of the regression line, the r^2^ and the number of all listed CRs are reported in Table 1, separately for the two examined calendar periods (1983–1987 and 2008–2012). A statistically significant direct linear relationship between median age at cancer onset and median age of the corresponding population was observed by the majority of examined cancers, although the values of the slopes varied.

In males, the all sites but skin group in 2008–2012 showed a slope of 0.45 (95% confidence interval, CI: 0.41–0.49; *p* < 0.01, i.e., the median age at cancer onset increased nearly ½ year for each one-year increase in population ageing). Through the linear model, a 56% percent of the variance of the median age of cancer onset was explained by the variance of population ageing (r^2^ = 0.56). In females, the slope was 0.62 (95% CI: 0.57–0.66) with a r^2^ = 0.65, i.e., the median age at cancer onset increased nearly 2/3 of a year for each one-year increase of median population ageing, and the variability explained by the linear model was 65%. The slope was steeper in women than in man.

When considering specific cancers or groups of cancers, the median values of all calculated slopes was 0.41 in males and 0.57 in females. In particular, in males, the highest values of the slopes were observed for leukemias (1.47), non-Hodgkin disease (0.79), Hodgkin disease (0.64), and kidney cancer (0.63), while in females, for leukemias (1.50), kidney cancer (0.90), non-Hodgkin disease (0.80), and stomach cancer (0.67). The lowest values of the slopes were observed in males for the cancers of prostate (0.10), larynx (0.21), esophagus (0.22), and testis (0.24), while in females, the lower values were observed for cervix uteri cancer (0.07), larynx cancer (0.26), lung cancer (0.30), and Hodgkin disease (0.36). The median of all r^2^ values was 0.42 (range: 0.04–0.59) in males and 0.45 (range: 0.02–0.67) in females.

The slopes of the 22 cancers or group of cancers in the two periods (1983–1987 and 2008–2012) were compared by means of the Spearman correlation coefficient. The coefficient was 0.92 in males and 0.89 in females, indicating that the slopes tended to maintain a similar pattern across the two periods.

The association between the median age of the population and the median age at cancer onset was further disentangled by the three selected broad geographical areas (Americas, Europe, and Oceania; Asia; Eastern Mediterranean and Africa) during the 2008–2012 period (Table S1 and Figure 1a,b). Visually, the slopes of the two main geographical locations displaying the higher number of CRs (i.e., all but African area that consisted of 24 CRs: 6.1% of the total) matched in the majority of cancers or groups. The Spearman correlation coefficient of the slopes of the 22 cancers or group of cancers was computed to mutually compare the three areas. The coefficients ranged from 0.67 to 0.73 in males and from 0.79 to 0.90 in females. As a sensitivity analysis, we recalculated models for the 2008–2012 period, excluding the 109 CRs that did not fully meet all quality criteria of IARC (i.e., we analyzed 284 CRs instead of 393). For all cancers, but skin, no relevant differences in the slopes were noted in both sexes: in males 0.45 (95% CI: 0.41–0.49) vs. 0.42 (95% CI: 0.37–0.47), and in females 0.62 (95% CI: 0.57–0.66) vs. 0.59 (95% CI: 0.52–0.65) for the 393 CRs and the 284 CRs, respectively.

Finally, to validate the association between the median age of the population and the median age at cancer onset, we considered only the CRs that recorded data for approximately 25 years (the so-called long-standing CRs). For each cancer or group of cancers, a 2-point line graph displayed the median age at cancer onset according to the median age of the underlying populations in each long-standing CR at the beginning (1983–2087) and at the end (2008–2012) of the study period, in males and females separately (Supplementary Figure S1a,b, respectively).

The pattern observed was different among cancers or groups of cancers. After 25-year, for all cancers but skin the increase of the median age at cancer onset was 1.1 years (range: −4.9–+8.2) in males, and +0.9 years (−2.0–+8.8) in females. In about 1/3 of the CRs, the two medians decreased after 25 years in both sexes. This pattern was particularly evident when the median age of the population tended to be elevated in 1983–1987. In males, a ROC curve analysis showed that an age cut-off at cancer onset of 61.8 years in 1983–1987 predicted the decrease of the medians after 25-year (results available upon request to the corresponding author) whereas the cut-off in females was 66.7 years. The area under the curve was 0.74 in males and 0.82 in females. The majority of cancers or groups of cancer showed a coherent increase of the two medians after 25 years in both sexes. However, two decreases in the median age at cancer onset were observed, one for prostate cancer (−5.6 years; range: −7.8–+2.9) and one for cervix cancer (−2.1 years; range: −14.6–+11.0).

## 4. Discussion

Our observational study examined incidence data from 20 cancer types/sites derived from validated worldwide CRs data gathered in 25 years. The findings highlighted that the median age at cancer onset was linearly proportional to the median age of the underlying population at risk. This linear association was similar in the two main geographical locations displaying the higher number of CRs considered (i.e., all but African area) in the majority of cancers or groups. The contribution of age to the linear pattern observed in all cancers but skin was around 50%. Conversely, the slopes of the linear regression and the variability explained by the model changed according to cancer sites and geographical areas. Finally, when examining the long-standing CRs, in almost all examined cancers but prostate and cervix uteri, the median age at cancer onset at the beginning of the study (i.e., 1983–1987) tended to increase after 25 years (2008–2012) correspondingly with the ageing of the population. The observed pattern firstly gave insights on the worldwide diagnostic changes occurred for prostate and cervical cancers during the examined period, and secondly that for most types of cancer, a younger age structure of a population is linked to a high frequency of young age at cancer onset, whereas an older age structure of a population is linked to a higher frequency of older age at cancer onset. Consequently, since age at cancer onset is embedded with the age structure of the underlying population, it is possible to avoid the misinterpretation of a high frequency of cancer onset in specific age groups by simply inspecting the age pyramid of the population in order to check the plausibility of the excess.

The linear patterns observed are broadly similar to the findings from two previous works on BC site [6,7] and in line with the investigations that described the increase of age-specific mortality rates with ageing in many forms of cancers [4,5]. However, besides considering the linear patterns of the associations, the wide range of the variability explained by the linear models in the various cancer sites examined may be elucidated by means of several plausible biological mechanisms, medical interventions, or early diagnostic procedures. These factors can lead to a modification of the natural course of the cancer diagnosis in some sites, conducting to the elevated variability observed.

It is known that some cancer sites are more prone to multiple primary cancers than others, and that the second primary cancers may be conditional upon the first cancer [17,18,19,20]. For instance, the exposure to the agents which caused the primary cancers may trigger the development of multiple primary cancers in etiologically related anatomical sites, during a process defined sometimes as “field cancerization” theory. Additionally, cancer survival has increased throughout years [21] and cancer survivors became generally at risk of developing second primary cancer later in life because of ageing, of long-term sequelae of therapies, or of careful patient’s surveillance over a long period that increased the probability of detection of new cancers [22,23,24]. Moreover, in some sites, it may be difficult to distinguish a second primary cancer from an extension from an adjacent site, a recurrence of a first cancer or metastases. Likewise, the criteria for defining second primary cancers have changed over time [18] and actually two sets of rules are widely used according to CRs, the rules of the Surveillance Epidemiology and End Results (SEER) Program [25] that are used mainly by North American CRs, and the rules developed by the International Agency for Research on Cancer (IARC) [26]. In particular, the IARC rules are more exclusive as only one cancer is recorded for each organ (or organ pair), regardless of time and morphological homologous cancers must be counted only once [18].

The variance explained by the linear model was poor in head and neck (pharynx and larynx) and esophageal cancers, especially in men. Besides the difficulty in identifying the primary site of cancer onset leading to its potential anatomical misclassification [25,26], head and neck cancers have also a high probability of being followed by second primary cancers, which are located anatomically most commonly near to the primary tumor (i.e., in head and neck or esophagus), the so-called field cancerization [21]. Observed results likely reflected the difficulties linked to the definition of the primary anatomical site and/or to the presence of metachronous secondary sites conditional to the first cancer.

The introduction of prostate-specific antigen (PSA) testing in the late 1980s substantially increased prostate cancer incidence rates [27]. Concerns about overscreening and overdiagnosis subsequently led professional guidelines to no longer recommend PSA testing for men at average risk of prostate cancer [28,29]. During the 25-year span of our study, we showed a decrease in the median age at prostate cancer onset correspondingly to the ageing of the underlying population. Since the introduction of an early detection practice anticipates the diagnosis of cancers, and consequently lowers the age at diagnosis by concentrating the diagnoses in specific age ranges, it is likely that the pattern observed in our study is linked to the worldwide advent of PSA testing.

Cervical cancer showed the same temporal pattern of prostate cancer throughout the 25-year of observation. Generally, a population screening lead to the diagnosis of cervical cancers before the beginning of symptoms and subsequently lowered the age at cancer diagnosis in the specific age range of the targeted population. Our results showed a worldwide decrease in the median age at cancer onset with a corresponding increase of the ageing of the population, supporting the advance in cervical cancer control during the studied 25-year span.

It should be noted that the median age of the population explained the age variance at cancer diagnosis less in the Eastern Mediterranean and Africa area than in the other two geographical areas. A possible reason for this pattern is the relatively low number of CRs examined in this area. This pattern may also be explained by the higher awareness raised about primary and secondary cancer prevention that led to a more homogeneous behavior concerning age at diagnosis [30]. Another possible explanation is the full epidemiological transition raised in the Americas, Europe, Oceania, and Asia areas that shifted globally the burden of disease from mainly infectious diseases to chronic non-communicable diseases [31].

In any case, although our linear model tended to indicate that nearly half of BC cases were explained by population ageing, the burden of young women with early BC justify public awareness campaigns towards early detection (possibly population-based), according to International directives [32], especially in young populations regardless of income of the country of living (i.e., LMIC but also in HIC). Moreover, public awareness about BC risk factors is another key point to consider.

This study presented several strengths. First, the study used all the data compiled by IARC on the worldwide cancer incidence and selected the subset of CRs that gathered long-term incidence data (i.e., for a 25-year span). Second, by definition the analyzed CRs met international standards for validation, high-quality and comparability of the methodology of registration. Moreover, CRs guaranteed the complete coverage of the resident population. Third, the grouping of CRs in three main geographical areas helped the interpretation of the studied age patterns. Furthermore, it is likely that the sample was sufficiently large to limit faulty interpretation due to random variations. Fourth, the variability of the pyramids of age displayed by CRs gave clues to the interpretation of this investigation. Fifth, the change in international classification of the examined cancer sites or groups (IX to X revision) is unlikely to present classification issues between the two revisions.

By contrast, the study suffered from limitations that are common to other observational studies. First, causal inferences on unexplained variability was limited by the impossibility to gather information about individual risk factors on cancers, i.e., lifestyle, nutritional, reproductive habits, and medical history, and the use of screening tests. Second, we assumed a linear relationship between the median age of the population and the median age at cancer onset by examining merely their scatterplot and analytically excluding the exponential model. Furthermore, all medians were calculated from the matrices of quinquennia of age of the CRs and the corresponding population, thus these estimates may be imprecise in the presence of CRs with small populations. Third, the generalization of our results at country level must be exercised with caution when CRs have a small base population. Finally, part of the variability of the median age at cancer onset was explained by the median age of the population worldwide. Thus, the residual unexplained variability by the linear model may reflect the proportion attributable to the specific risk factors associated with the studied cancers and the presence of multiple cancers in the dataset.

## 5. Conclusions

In conclusion, our work showed that the linear relationship between the two analyzed ages could be hypothesized in the majority of cancer groups and in some geographical areas. By using public data sources, firstly, this approach pinpointed some diagnostic changes occurred throughout the studied 25-year span. Secondly, the plausibility of high frequencies of cancer onset in specific age groups can be checked by the examination of the age structure of the underlying population.

## Figures and Tables

**Table 1:** Linear regression between median age of the population as a predictor of the median age of the studied cancers according to two periods (1983–1987 and 2008–2012) recorded by the Cancer in Five Continents volumes (VI and XI, respectively) and sex.

		1983–1987 (Volume VI, *N* = 160 CRs)				2008–2012 (Volume XI, *N* = 393 CRs)			
Cancer Site or Groups	ICD-10	r^2^	α	Slope	(95%CI)	r^2^	α	Slope	(95%CI)
Males									
All sites, but skin	C00–96, but C44	0.42+	49.8	0.52 *	(0.43–0.62)	0.56+	50.4	0.45 *	(0.41–0.49)
Pharynx	C09–C14	0.13+	50.3	0.32 *	(0.19–0.44)	0.19+	49.2	0.31 *	(0.25–0.38)
Oesophagus	C15	0.11+	58.3	0.24 *	(0.13–0.35)	0.14+	58.6	0.22 *	(0.17–0.28)
Stomach	C16	0.36+	54.2	0.45 *	(0.36–0.54)	0.47+	51.8	0.47 *	(0.42–0.52)
Liver	C22	0.27+	49.6	0.52 *	(0.38–0.65)	0.24+	51.4	0.39 *	(0.32–0.45)
Pancreas	C25	0.24+	54.5	0.42 *	(0.30–0.53)	0.42+	53.4	0.41 *	(0.36–0.46)
Colon, rectum, and anus	C18–21	0.42+	51.8	0.51 *	(0.42–0.60)	0.56+	48.8	0.52 *	(0.47–0.56)
Larynx	C32	0.13+	55.9	0.22 *	(0.13–0.31)	0.18+	57.3	0.21 *	(0.17–0.26)
Lung	C33–34	0.27+	57.0	0.29 *	(0.22–0.37)	0.39+	56.7	0.34 *	(0.30–0.38)
Prostate	C61	0.19+	68.8	0.16 *	(0.11–0.21)	0.04+	66.1	0.10 *	(0.05–0.15)
Testis	C62	0.07+	26.9	0.20 *	(0.08–0.32)	0.07+	26.4	0.24 *	(0.16–0.33)
Bladder	C67	0.23+	60.6	0.26 *	(0.19–0.33)	0.37+	58.3	0.35 *	(0.30–0.39)
Kidney	C64–66, C68	0.36+	37.4	0.82 *	(0.65–0.99)	0.45+	41.2	0.63 *	(0.56–0.70)
Hodgkin lymphoma	C81	0.18+	17.8	0.70 *	(0.47–0.94)	0.17+	18.3	0.64 *	(0.50–0.79)
Non-Hodgkin lymphoma	C82–85, C96	0.52+	28.1	1.03 *	(0.87–1.19)	0.59+	34.6	0.79 *	(0.72–0.85)
Myeloma	C88, 90	0.24+	54.5	0.41 *	(0.30–0.53)	0.36+	50.5	0.48 *	(0.42–0.55)
Leukaemias	C91–95	0.52+	3.3	1.79 *	(1.52–2.06)	0.53+	7.9	1.47 *	(1.33–1.60)
Ill-defined or unspecified						0.44+	47.6	0.62 *	(0.55–0.69)
Females									
All sites, but skin	C00–96, but C44	0.65+	41.7	0.68 *	(0.60–0.75)	0.65+	40.3	0.62 *	(0.57–0.66)
Pharynx	C09–C14	0.21+	43.6	0.52 *	(0.36–0.67)	0.17+	41.3	0.49 *	(0.39–0.60)
Oesophagus	C15	0.29+	52.9	0.54 *	(0.41–0.68)	0.27+	52.0	0.49 *	(0.41–0.57)
Stomach	C16	0.45+	51.3	0.61 *	(0.51–0.72)	0.49+	44.9	0.67 *	(0.60–0.74)
Liver	C22	0.41+	47.6	0.66 *	(0.54–0.79)	0.45+	46.2	0.63 *	(0.56–0.70)
Pancreas	C25	0.41+	55.0	0.49 *	(0.40–0.58)	0.48+	50.6	0.57 *	(0.51–0.63)
Colon. rectum and anus	C18–21	0.48+	48.9	0.62 *	(0.52–0.72)	0.67+	43.8	0.67 *	(0.62–0.71)
Larynx	C32	0.08+	54.8	0.25 *	(0.11–0.39)	0.08+	54.7	0.26 *	(0.17–0.35)
Lung	C33–34	0.37+	55.2	0.35 *	(0.28–0.42)	0.31+	57.1	0.30 *	(0.26–0.35)
Breast	C61	0.45+	41.7	0.53 *	(0.44–0.62)	0.42+	41.1	0.47 *	(0.42–0.53)
Cervix uteri	C62	0.32+	38.5	0.46 *	(0.35–0.56)	0.02+	48.3	0.07 *	(0.02–0.13)
Bladder	C67	0.33+	51.4	0.58 *	(0.46–0.71)	0.26+	56.3	0.42 *	(0.35–0.49)
Kidney	C64–66, C68	0.42+	35.3	0.87 *	(0.71–1.03)	0.47+	31.1	0.90 *	(0.80–0.99)
Hodgkin lymphoma	C81	0.11+	22.1	0.47 *	(0.26–0.67)	0.07+	24.7	0.36 *	(0.23–0.48)
Non-Hodgkin lymphoma	C82–85, C96	0.54+	31.5	0.98 *	(0.84–1.12)	0.58+	34.7	0.80 *	(0.73–0.87)
Myeloma	C88, 90	0.41+	52.3	0.52 *	(0.42–0.62)	0.45+	46.8	0.59 *	(0.52–0.65)
Leukaemias	C91–95	0.56+	-0.1	1.81 *	(1.56–2.06)	0.61+	4.9	1.50 *	(1.38–1.62)
Ill-defined or unspecified						0.53+	42.2	0.83 *	(0.75–0.90)

CR: Cancer Registry; α: Intercept; ICD-10: International Classification of Diseases. X Revision; r^2^: R-squared; slope: slope of the regression line; 95% CI: 95% Confidence Interval; + probability of *F*-test < 0.01; * *p* < 0.01.

## Data Availability

The datasets analyzed during the current study were downloaded from the IARC website: http://ci5.iarc.fr (accessed on 28 October 2021). and the currently analyzed data are available from the corresponding author upon reasonable request.

## Supplementary Materials

The following are available online at https://www.mdpi.com/article/10.3390/cancers13215589/s1, Figure S1: Vector plots of the median age of the population and the median age at cancer diagnosis at the beginning (1983–87) and at the end (2008–12) of the studied period in the 84 populations of the long-standing cancer registries (CRs) listed in the Cancer in Five Continents volumes. (a) Males; (b) Females, Table S1: Linear regression between median age of the population as a predictor of the median age of the studied cancers according to the three selected main geographical areas recorded by the XI Cancer in Five Continents volumes (2008–12).
