# Supplementary material for: Linearity of Age at Cancer Onset Worldwide: 25-Year Population-Based Cancer Registry Study
Source: Cancers (Basel). 2021 Nov 8;13(21):5589. doi: 10.3390/cancers13215589 (PMC8583131; doi:10.3390/cancers13215589)
Supplement: Supplementary file 1 [file cancers-13-05589-s001.zip › cancers-1431313-supplementary.pdf]

Table S1: Linear regression between median age of the population as a predictor of the median age of the studied cancers according to the three selected main geographical areas recorded by the XI Cancer in Five Continents volumes (2008-12)

	Selected main geographical areas											
	Americas, Europe and Oceania (N= 285 CRs)				Asia (N= 84 CRs)				Eastern Mediterranean and Africa (N= 24 CRs)			
**Cancer**	r^2	α	Slope	(95%CI)	r^2	α	Slope	(95%CI)	r^2	α	Slope	(95%CI)
Males												
All sites, but skin	0.61+	53.9	0.38*	(0.34;0.41)	0.67+	45.8	0.53*	(0.45;0.61)	0.10	53.0	0.30	(0.08;0.68)
Pharynx	0.14+	53.7	0.21*	(0.15;0.28)	0.04	53.4	0.16	(0.01;0.33)	0.06	46.5	0.20	(0.01;0.54)
Oesophagus	0.09+	60.3	0.19*	(0.12;0.25)	0.28+	53.7	0.32*	(0.21;0.44)	0.00	63.9	0.03	(0.36;0.41)
Stomach	0.53+	52.6	0.47*	(0.42;0.52)	0.51+	50.3	0.43*	(0.34;0.52)	0.03	60.5	0.13	(0.20;0.46)
Liver	0.31+	50.4	0.43*	(0.36;0.51)	0.14+	52.3	0.27*	(0.12;0.42)	0.03	57.2	0.20	(0.30;0.70)
Pancreas	0.41+	56.1	0.35*	(0.30;0.40)	0.58+	45.2	0.58*	(0.48;0.69)	0.03	61.0	0.14	(0.20;0.47)
Colon and rectum	0.59+	51.5	0.46*	(0.42;0.51)	0.62+	46.1	0.52*	(0.43;0.61)	0.21	51.1	0.38	(0.07;0.70)
Larynx	0.09+	59.7	0.15*	(0.10;0.21)	0.34+	53.3	0.32*	(0.22;0.42)	0.04	63.9	-0.09	(-0.28;0.09)
Lung	0.31+	59.5	0.28*	(0.23;0.32)	0.61+	52.0	0.45*	(0.37;0.53)	0.00	65.3	-0.04	(-0.36;0.28)
Prostate	0.15+	61.7	0.19*	(0.14;0.24)	0.21+	66.6	0.19*	(0.11;0.26)	0.24	75.9	-0.19*	(-0.33;-0.05)
Testis	0.20+	25.9	0.24*	(0.18;0.29)	0.05+	26.3	0.33*	(0.01;0.64)	0.01	28.8	0.13	(0.46;0.72)
Bladder	0.31+	61.0	0.29*	(0.24;0.34)	0.48+	55.4	0.38*	(0.30;0.47)	0.01	64.4	0.05	(0.24;0.34)
Kidney	0.54+	47.1	0.50*	(0.45;0.54)	0.46+	44.0	0.49*	(0.38;0.61)	0.29	15.6	1.46*	(0.51;2.40)
Hodgkin lymphoma	0.10+	24.9	0.42*	(0.27;0.57)	0.35+	1.9	1.27*	(0.89;1.65)	0.13	18.4	0.56	(0.05;1.16)
Non-Hodgkin lymphoma	0.54+	40.3	0.66*	(0.59;0.73)	0.62+	35.4	0.70*	(0.58;0.81)	0.46	24.2	1.02*	(0.56;1.48)
Myeloma	0.44+	50.5	0.50*	(0.43;0.57)	0.26+	48.9	0.46*	(0.29;0.63)	0.01	65.0	-0.07	(-0.43;0.29)
Leukaemias	0.56+	18.7	1.25*	(1.12;1.38)	0.77+	-9.5	1.68*	(1.48;1.87)	0.30	8.9	1.40*	(0.51;2.29)
Ill-defined or unspecified	0.51+	49.7	0.60*	(0.53;0.66)	0.42+	44.2	0.61*	(0.45;0.77)	0.07	54.9	0.33	(0.16;0.81)
Females												
All sites, but skin	0.73+	45.7	0.51*	(0.47;0.54)	0.60+	37.2	0.62*	(0.51;0.73)	0.33	41.5	0.49	(0.30;0.79)
Pharynx	0.09+	45.3	0.42*	(0.26;0.57)	0.15+	45.2	0.32*	(0.16;0.49)	0.10	41.6	0.34	(0.01;0.77)
Oesophagus	0.20+	51.8	0.50*	(0.38;0.61)	0.54+	48.4	0.56*	(0.45;0.67)	0.00	65.3	0.03	(0.39;0.45)
Stomach	0.45+	49.5	0.59*	(0.51;0.66)	0.60+	39.7	0.69*	(0.57;0.82)	0.18	51.2	0.43	(0.05;0.81)
Liver	0.40+	46.2	0.64*	(0.55;0.73)	0.53+	43.6	0.63*	(0.50;0.76)	0.35	48.7	0.62*	(0.27;0.96)
Pancreas	0.44+	57.6	0.41*	(0.36;0.47)	0.48+	41.9	0.72*	(0.56;0.89)	0.45	49.4	0.57*	(0.31;0.83)
Colon and rectum	0.66+	48.3	0.58*	(0.53;0.62)	0.76+	39.2	0.71*	(0.62;0.79)	0.41	45.5	0.58*	(0.29;0.87)
Larynx	0.05+	57.1	0.19*	(0.09;0.28)	0.19+	45.7	0.54*	(0.30;0.78)	0.03	67.6	-0.23	(-0.78;0.32)
Lung	0.13+	62.3	0.18*	(0.13;0.23)	0.64+	47.6	0.54*	(0.45;0.62)	0.00	65.4	-0.04	(-0.29;0.21)
Breast	0.63+	46.1	0.39*	(0.35;0.42)	0.33+	42.0	0.30*	(0.21;0.39)	0.18	44.7	0.26*	(0.03;0.49)
Cervix uteri	0.08+	43.3	0.20*	(0.12;0.28)	0.03+	54.1	-0.07	(-0.28;0.25)	0.00	52.3	-0.02	(-0.28;0.25)
Bladder	0.12+	62.0	0.29*	(0.20;0.38)	0.46+	47.4	0.61*	(0.47;075)	0.12	60.0	0.25	(0.03;0.54)
Kidney	0.52+	46.4	0.54*	(0.48;0.60)	0.52+	22.2	1.06*	(0.84;1.28)	0.34	-0.5	1.85*	(0.77;2.93)
Hodgkin lymphoma	0.03+	29.9	0.20*	(0.06;0.35)	0.13+	18.7	0.61*	(0.26;0.96)	0.18	19.7	0.40*	(0.05;0.76)
Non-Hodgkin lymphoma	0.56+	42.6	0.63*	(0.56;0.70)	0.60+	32.4	0.76*	(0.63;0.89)	0.51+	20.7	1.21*	(0.72;1.71)
Myeloma	0.49+	50.9	0.51*	(0.45;0.57)	0.37+	41.6	0.62*	(0.45;0.80)	0.27	50.5	0.45*	(0.14;0.76)
Leukaemias	0.60+	16.5	1.27*	(1.15;1.39)	0.75+	-3.6	1.49*	(1.30;1.67)	0.50	-1.7	1.7*	(0.99;2.40)
Ill-defined or unspecified	0.55+	47.9	0.72*	(0.64;0.79)	0.52+	35.9	0.85*	(0.67;1.03)	0.54+	39.3	0.95*	(0.58;1.33)

α: Intercept; CR: Cancer Registry;
r^2: R-squared; slope: slope of the regression line; 95%CI: 95% Confidence Interval; +probability of F-test<0.01; *p<0.01

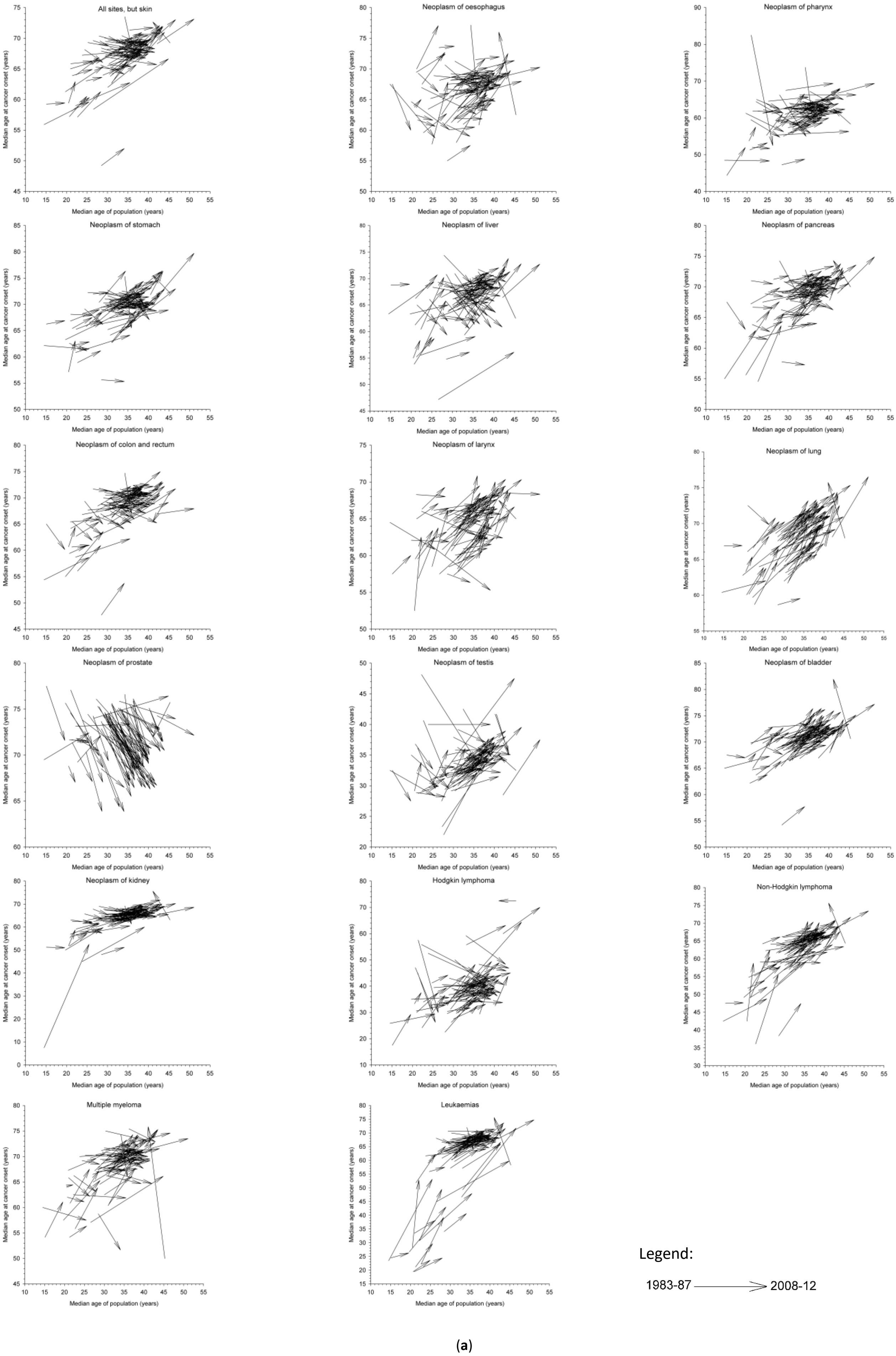
All sites, but skin
Neoplasm of oesophagus
Neoplasm of pharynx
Neoplasm of stomach
Neoplasm of liver
Neoplasm of pancreas
Neoplasm of colon and rectum
Neoplasm of larynx
Neoplasm of lung
Neoplasm of prostate
Neoplasm of testis
Neoplasm of bladder
Neoplasm of kidney
Hodgkin lymphoma
Non-Hodgkin lymphoma
Multiple myeloma
Leukaemias
Median age at cancer onset (years)
Median age of population (years)
Legend:
1983-87
2008-12

(a)

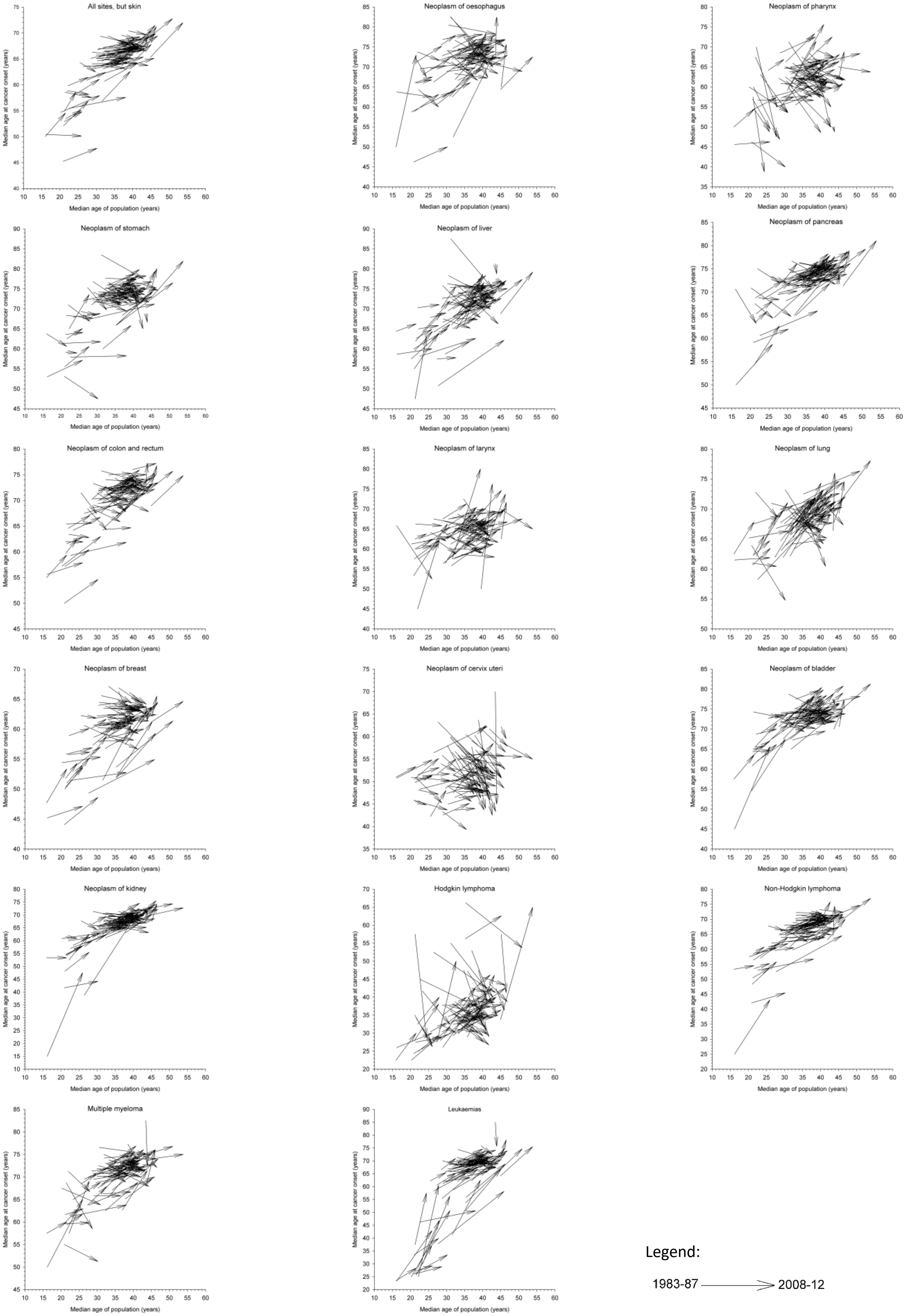
All sites, but skin
Neoplasm of oesophagus
Neoplasm of pharynx
Neoplasm of stomach
Neoplasm of liver
Neoplasm of pancreas
Neoplasm of colon and rectum
Neoplasm of larynx
Neoplasm of lung
Neoplasm of breast
Neoplasm of cervix uteri
Neoplasm of bladder
Neoplasm of kidney
Hodgkin lymphoma
Non-Hodgkin lymphoma
Multiple myeloma
Leukaemias
Median age at cancer onset (years)
Median age of population (years)
Legend:
1983-87
2008-12

(**b**)

Figure S1. Vector plots of the median age of the population and the median age at cancer diagnosis at the beginning (1983-87) and at the end (2008-12) of the studied period in the 84 populations of the long-standing cancer registries (CRs) listed in the Cancer in Five Continents volumes. (**a**) Males; (**b**) Females.
